# Effects of dry and traditional bed bathing on respiratory parameters: a randomized pilot study

**DOI:** 10.1590/1518-8345.3668.3264

**Published:** 2020-06-01

**Authors:** Luana Vieira Toledo, Patrícia de Oliveira Salgado, Cristiane Chaves de Souza, Lídia Miranda Brinati, Carla de Fátima Januário, Flávia Falci Ercole

**Affiliations:** 1 Universidade Federal de Viçosa, Departamento de Medicina e Enfermagem, Viçosa, MG, Brasil.; 2 Universidade Federal de Minas Gerais, Escola de Enfermagem, Belo Horizonte, MG, Brasil.; 3 Hospital São Sebastião, Unidade de Terapia Intensiva, Viçosa, MG, Brasil.

**Keywords:** Baths, Nursing, Oximetry, Critical Care, Intensive Care Units, Respiratory Rate, Banhos, Enfermagem, Oximetria, Cuidados Críticos, Unidades de Terapia Intensiva, Frequência Respiratória, Banhos, Enfermagem, Oximetria, Cuidados Críticos, Unidades de Terapia Intensiva, Frecuencia Respiratoria

## Abstract

**Objective:**

to compare the time for performance of dry and traditional bed bathing and its effects on transcutaneous arterial oxygen saturation and respiratory rates in critical adult patients.

**Method:**

pilot study of a randomized, open, crossover clinical trial, performed with 15 adult critically ill patients. Each patient received a dry and a traditional bed bath. Analysis of variance with repeated measures was used, adopting p-value ≤ 0.05.

**Results:**

most patients were male (73.3%), white (66.7%), with a mean age of 69.7 years. The dry bath was faster (20.0 minutes) than the traditional bath (30.0 minutes) (p<0.001). There was no significant difference between the patients’ saturation means between baths (p=0.381), with 94.7% for the dry bath and 95.2% for the traditional bath. During the traditional bath, the patients’ respiratory rate mean was higher (24.2 incursions *per* minute) and statistically different (p<0.001) from the value obtained for the dry bath (20.5 incursions *per* minute).

**Conclusion:**

the dry bath had a shorter duration than did the traditional bath, resulting in less patient exposure. The traditional bed bath had a negative effect on patients’ respiratory rate, increasing it. Brazilian Registry of Clinical Trials (ReBEC): RBR-5qwkqd

## Abstract (pt)

**Objetivo:**

comparar o tempo de execução do banho no leito a seco e do tradicional, e os seus efeitos sobre a saturação transcutânea de oxigênio arterial e a frequência respiratória em pacientes críticos adultos.

**Método:**

estudo piloto de um ensaio clínico randomizado *crossover*, aberto, realizado com 15 pacientes críticos adultos. Cada paciente recebeu o banho no leito a seco e o tradicional. Utilizou-se a análise de variância com medidas repetidas, adotando-se o valor p≤0,05.

**Resultados:**

a maioria dos pacientes era do sexo masculino (73,3%), brancos (66,7%), com média de idade de 69,7 anos. O banho a seco foi mais rápido (20,0 minutos) que o tradicional (30,0 minutos) (p<0,001). Não houve diferença significativa entre as médias de saturação dos pacientes entre os banhos (p=0,381), sendo 94,7% no banho a seco e 95,2% no tradicional. Durante o banho tradicional, a média da frequência respiratória dos pacientes foi maior (24,2 incursões por minuto) e estatisticamente diferente (p<0,001) do valor obtido no banho a seco (20,5 incursões por minuto).

**Conclusão:**

o banho a seco teve uma duração menor que o tradicional, resultando em uma menor exposição dos pacientes. O tradicional banho no leito gerou efeito negativo sobre a frequência respiratória dos pacientes, elevando-a. Registro Brasileiro de Ensaios Clínicos (ReBEC): RBR-5qwkqd

## Abstract (es)

**Objetivo:**

comparar el tiempo de ejecución del baño en la cama a seco y con el tradicional, y observar los efectos sobre la saturación del oxígeno transcutáneo arterial y la frecuencia respiratoria, en pacientes críticos adultos.

**Método:**

estudio piloto de un ensayo clínico aleatorizado *crossover*, abierto, realizado con 15 pacientes críticos adultos. Cada paciente recibió el baño en la cama, a seco y de forma tradicional. Se utilizó el análisis de variancia con medidas repetidas adoptando el valor p≤0,05.

**Resultados:**

la mayoría de los pacientes era del sexo masculino (73,3%), blancos (66,7%), con media de edad de 69,7 años. El baño a seco fue más rápido (20,0 minutos) que el tradicional (30,0 minutos) (p<0,001). No hubo diferencia significativa entre los promedios de saturación de los pacientes entre los baños (p=0,381), siendo 94,7% en el baño a seco y 95,2% en el tradicional. Durante el baño tradicional la media, de la frecuencia respiratoria, de los pacientes fue mayor (24,2 incursiones por minuto) y estadísticamente diferente (p<0,001) del valor obtenido en el baño a seco (20,5 incursiones por minuto).

**Conclusión:**

El baño a seco tuvo una duración menor que el tradicional, resultando en una menor exposición de los pacientes. El tradicional baño en la cama generó efecto negativo sobre la frecuencia respiratoria de los pacientes, elevándola. Registro Brasileño de Ensayos Clínicos (ReBEC): RBR-5qwkqd

## Introduction

In Intensive Care Units (ICUs), patients can often lose their autonomy for self-care, experiencing the feeling of helplessness while attempting to meet basic human needs, such as keeping personal hygiene^(^ 1 ^)^.

Most of these patients are unable to perform their bath by themselves, and it is up to the nursing team to do so, in the form of a bed bath^(^ 2 ^)^. This nursing practice is considered a therapeutic option that provides clinical benefits to patients, such as stimulating circulation, inducing comfort and relaxation^(^ 3 ^-^ 4 ^)^.

However, bed bathing can also generate risks to patient safety, such as risk for infection, bed falls and displacement of patient-care devices^(^ 5 ^)^. It is also evident that prolonged baths, lasting more than 20 minutes, are considered as a risk factor for changes in Transcutaneous Arterial Oxygen Saturation (SpO_2_); body temperature; Blood Pressure (BP); Heart Rate (HR) and Respiratory Rate (RR)^(^ 6 ^)^.

With regard specifically to the effects of bathing on respiratory parameters, a study conducted with critically ill cardiovascular patients showed the occurrence of tachypnea in 66.7% of patients, which may be related to their handling during the procedure^(^ 7 ^)^. A similar result was found in a study carried out in Medellin, in which critical patients showed significant RR increase (p<0.0001) after the traditional bed bath^(^ 8 ^)^. SpO2 values, in turn, remained above 95% and were not statistically different (p=0.472) throughout the bath^(^ 8 ^)^.

In view of the above, the performance of the traditional bed bath has been questioned in the scientific community^(^ 9 ^-^ 10 ^)^. In this context, in order to minimize the risks of traditional bed bathing, a new bathing method, known as bag bath, dry bath or disposable bath, has been proposed. In this new type of bed bath, disposable cotton wipes are used. They are pre-moistened in an emollient solution and intended for cleaning a body area, which, after cleaning, does not require rinsing or drying^(^ 11 ^)^. Researchers have dedicated themselves to assessing the acceptability by patients and professionals in relation to this type of bath, which has shown positive results^(^ 12 ^)^. However, as it is still a recent practice in many care settings, dry bathing should be seen as an object of study in nursing, in search of scientific evidence that supports it as a safe and effective bed bathing practice.

The dry bed bath has been considered a promising alternative to the traditional bed bath due to lower risk for skin recontamination, lower cost and shorter time of performance^(^ 13 ^-^ 14 ^)^. However, in addition to such observed advantages, it is necessary to consider the effect of this new bathing method on patients’ respiratory parameters, especially those related to SpO2 and RR. For critically ill patients, these variables can be an important indicator that precedes the clinical manifestation of serious complications and even worse prognoses^(^ 15 ^)^.

Despite the clinical importance of changes in respiratory parameters (SpO_2_ and RR) generated by bed bathing, there is a lack of studies aimed at evaluating them. There is a need to compare these respiratory parameters during the performance of the two types of bed baths (dry and traditional) in critically ill adult patients with different clinical conditions. From studies with high methodological rigor, it will be possible to show, between these two personal hygiene methods, the one that meets the real needs of critically ill patients, with a shorter performance time and less effect on respiratory parameters.

In view of the above, this pilot study was designed with the objective of comparing the time for performance of the dry and the traditional bed baths and their effects on SpO2 and RR in critically ill adult patients.

## Method

This is a pilot study of an open, randomized, crossover clinical trial (RCT), in which all participants received, at random, both interventions (dry and traditional bed bathing), and their SpO2 and RR values were recorded. Since it was a crossover study, the patient himself was considered his control. This study followed the recommendations by the Consolidated Standards of Reporting Trials (CONSORT)^(^ 16 ^)^.

This pilot study was conducted at the ICU of a university hospital, between the months of June and July 2018. The abovementioned ICU has six beds and, during the data collection period, 19 patients were admitted. The study population was composed of all patients hospitalized during this period.

The study included hospitalized patients aged 18 years or over who required the performance of the bed bath procedure for the purpose of promoting comfort and/or personal hygiene. Patients with severe burns and/or diarrhea were excluded. Patients who progressed to death, discharge or transfer before being subjected to the second bathing procedure were included in the discontinuity criterion.

The sample size followed the precepts recommended for pilot studies, in which a minimum number of 12 participants in each group is suggested^(^ 17 ^)^. Thus, the sample corresponded to all patients recruited in the period that met the inclusion criteria and who completed the follow-up, totaling 15 patients, who received both types of bed baths at random.

The randomization of each patient’s baths was performed by an independent researcher, external to the study, through the website *www.randomization.com*. A single-block randomization table was generated, with permutation of the two groups: intervention (dry bath) and control (traditional bath). After generating the randomization, the same external researcher distributed the random sequence of baths for each patient into sequential, numbered, opaque and sealed envelopes. The sequence of baths for each patient was performed according to randomization and, from there, the patients received, as the first bath, the dry bed bath or the traditional bath. The confidentiality of each patient’s allocation was disclosed to the researchers responsible for the study only when the procedure was performed, which was when the respective envelopes were opened.

After randomization, patients were submitted to the different types of bed baths (dry and traditional), the first bath being carried out in the first 24 hours of admission to the ICU. For each bath, there was a minimum interval of 24 hours (washout), in order to prevent the residual effect of one intervention on the other (carryover).

Data collection involved three people, the main researcher and two auxiliary researchers, one of whom was responsible for helping the main researcher with developing the bathing interventions, and the other being responsible for data recording. In order to participate in the study, eight auxiliary researchers underwent theoretical and practical training so as to ensure that the performance of interventions would be standardized. Interventions were performed according to the Standard Operating Procedure (SOP), taking as a reference the recommendations provided by the product manufacturer and the scientific literature^(^ 18 ^)^.

In the first training phase, the researchers received educational material on the method for bed bathing procedures and watched a video produced by the main researcher showing the completion of the steps for such procedures in a simulated environment. In the second phase, the auxiliary researchers, divided in pairs, reproduced the dry and traditional bed bathing techniques in a simulated environment and were evaluated as regards the fulfillment of phases from a check-list containing the steps necessary for proper performance of the technique, based on SOP^(^ 18 ^)^. To ensure the reliability of performance of the procedures, researchers were considered able to participate in the study if they achieved an Agreement Index greater than or equal to 0.9 in relation to the steps necessary to perform the two types of bed baths. The Agreement Index was evaluated using the formula: $\mathrm{AI}=\left(\mathrm{NA}/\mathrm{NA}+\mathrm{ND}\right)\times 100$, where NA is the number of agreements; and ND, the number of disagreements^(^ 19 ^)^. After two training cycles, all researchers were considered able to carry out the interventions.

The baths were performed uninterruptedly, but neither the patients’ oral cavities nor their scalps were cleaned during the procedures. The traditional bed bath included cleaning with soap and water, rinsing and body drying. The sequence of areas for body cleaning followed the cephalocaudal direction, starting with the region of the face, followed by the right and left upper limbs, trunk, right and left lower limbs and completing the anterior part by cleaning the genitals, going from the least contaminated to the most contaminated region. Then, the patients were turned to their sides in order to complete the cleaning of the dorsal part of the trunk and the gluteus. When patients were turned to their side, bed sheets were also replaced.

The dry bath was performed using individual packs of bath wipes produced by the FW - Feel Clean^®^ - wet wipes group. The procedure was performed according to SOP^(^ 18 ^)^. The sequence of body parts to be cleaned was the same as that for the traditional bed bath. It is noteworthy that, during the interventions, there was no blinding, as both researchers and patients, when lucid and oriented, knew the type of bath they should proceed with or receive.

The primary outcomes were the time taken to perform the dry bed bath and the traditional bed bath, as measured by a digital timer (Stopwatch^®^ ZSD-009) and recorded in minutes; SpO_2_, as measured from an adult oximetry sensor coupled to a multiparametric monitor (Dixtal^®^ Dx2023) and recorded in percentage (%); and RR, as measured by chest impedanciometry from the electrocardiogram electrodes of a multiparameter monitor (Dixtal^®^ Dx 2023) or a mechanical ventilator (Newport^®^ E 360br), whenever it was used, and recorded in respiratory incursions per minute (ripm). To compare the variation of outcomes, the variables related to the respiratory patterns (SpO_2_ and RR) were observed at fifteen minutes before beginning the bath; every five minutes during its performance and fifteen minutes after its completion. At the end, the mean values of each outcome in the procedures were obtained in order to identify the occurrence of statistically significant changes between them.

In addition to the primary outcomes, participants’ characterization data were recorded: age (years), sex (female/male), ethnicity (white, black, *pardo*, other), service responsible for referral to the ICU (emergency, internal medicine, surgery, others), cause of hospitalization (titles from the International Classification of Diseases - ICD-10), patient severity (Simplified Acute Physiology Score III - SAPS 3), associated comorbidities (nominal variable), medications and invasive devices used (nominal variable). It is noteworthy that other variables related to bed bathing, such as temperature (degrees Celsius) and humidity (percentage) in the ICU environment during the procedure, were also measured. To measure these variables, a digital thermo-hygrometer (Incoterm^®^ 7663) was used.

The data were double-entered into the Microsoft Office Excel program, version 2013, which was followed by descriptive and inferential analysis using the R-Bio program, version 107^(^ 20 ^)^. The Shapiro-Wilk test was applied to evaluate data normality. After normal distribution was verified, parametric tests were used. The participants’ characterization variables were compared using Fisher’s exact test. The mean time for bath performance, the temperature and humidity in the environment were evaluated using Student’s T test for paired samples. The mean values of the outcomes (RR and SpO_2_) and their 95% Confidence Intervals (CI) were analyzed by analysis of variance (ANOVA) with repeated measures. The alpha value of 5% probability was considered significant by Test F.

This study was approved by the Ethics and Research Committee of the promoting institution (Report no. 2.550.114) and registered on the Brazilian Clinical Trials Registry (ReBEC) platform under number RBR-5qwkqd. Patients who met the inclusion criteria were instructed as regards the objectives of the study and invited to participate in its conduction by signing an Informed Consent Form (ICF) and, whenever they were unable to do so, the study guidelines were presented to their legal representatives, who provided the required authorization.

## Results

**Figure 1:**
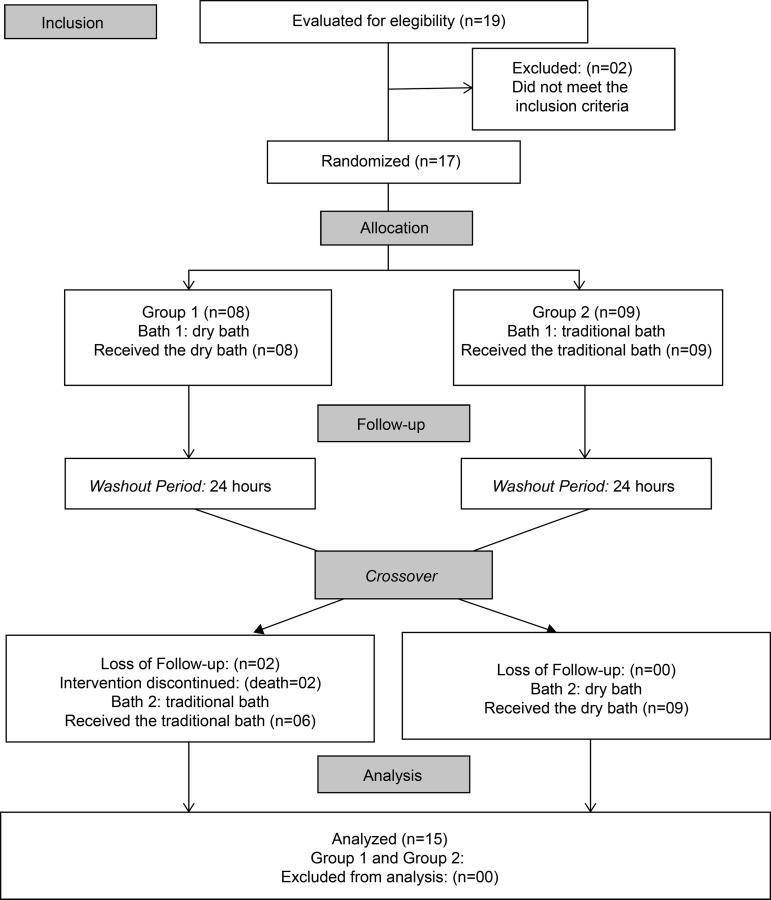
Flowchart of procedures for inclusion, allocation, follow-up and analysis of the study sample (n=15). Viçosa, MG, Brazil, 2018

During the study period, 19 patients were recruited, of whom two were excluded for not meeting the inclusion criteria, and two were included in the criteria for discontinuity, thus failing to complete the follow-up, as they evolved to death before performing the second bath. At the end, the sample consisted of 15 patients, as shown in Figure 1.

There was a predominance of patients showing the following characteristics: males (11 - 73.3%), white (10 - 66.7%), with a mean age of 69.7 years (± 14.5 years), referred to the ICU by the emergency service (9 - 60.0%), diagnosed with alterations in the respiratory system (7 - 46.7%), followed by alterations in the circulatory system (5 - 33.3%). The severity of patients was assessed by the Simplified Acute Physiology Score III (SAPS III), whose mean was 52.8 points (± 11.4), equivalent to a 22.8% probability of mortality (± 19.5). Regarding their previous pathological history, it was observed that 13 (86.7%) patients had associated comorbidities, especially high blood pressure (9 - 60.0%), diabetes *mellitus* (3 - 20.0%) and heart disease (3 - 20.0%), as described in Table 1.

**Table 1:** Sample characterization regarding sociodemographic and clinical variables on admission to the Intensive Care Unit (n=15). Viçosa, MG, Brazil, 2018

Variables		Patients (n=15)
Age M* (±SD^†^)	(Years)	69.7 (±14.5)
SAPS III^‡^ M*(±SD^†^)	Severity	52.80 (±11.4)
	Mortality Expectation	22.80 (±19.4)
Sex N^§^ (%)	Male	11 (73.3)
	Female	04 (26.7)
Race/Ethnicity N^§^ (%)	White	10 (66.7)
	Black	03 (20.0)
	*Pardo*	02 (13.3)
Origin N^§^ (%)	Emergency Services	09 (60.0)
	Internal Medicine	03 (20.0)
	Surgery Clinic	02 (13.3)
	Surgery	01 (6.7)
Diagnosis N^§^ (%)	Diseases of the respiratory system	07 (46.7)
	Diseases of the circulatory system	05 (33.3)
	Diseases of the genitourinary system	02 (13.3)
	Diseases of the digestive system	01 (6.7)
Comorbidities N^§^ (%)	Presence of comorbidities	13 (86.7)
Which comorbidities N^§^ (%)	High blood pressure	09 (60.0)
	Diabetes *Mellitus*	03 (20.0)
	Heart disease	03 (20.0)

* M = Mean;

† SD = Standard Deviation;

‡ SAPS III =Simplified Acute Physiology Score III;

§ N = Relative frequency

In general, during both the dry bath and the traditional bath, all patients were using analgesic drugs, and dipyrone was the most often used medication. Among invasive devices, it is noteworthy that 13 (86.7%) patients were using peripheral venous catheters during the dry bath, and 12 (80.0%) during the traditional bath. Oxygen therapy was present during both types of bath in 09 (60.0%) patients. The data regarding patients’ clinical conditions during the two baths did not show statistically significant differences, which represents homogeneity between the groups (Table 2).

**Table 2:** Clinical information of critically ill patients during the dry and traditional bed baths (n=15). Viçosa, MG, Brazil, 2018

Clinical Information	Dry bath (n=15)		Traditional bath (n=15)		p- value*
	N	%	n	%	
Medications					
Sedative Drugs	03	20.0	03	20.0	1.000
Vasoactive Drugs	07	46.7	06	40.0	0.713
Analgesic Drugs	15	100.0	15	100.0	1.000
Infusion Pump Drugs	12	80.0	13	86.7	0.624
Invasive Devices					
Peripheral Venous Catheter	13	86.7	12	80.0	0.624
Central Venous Catheter	03	20.0	03	20.0	1.000
Indwelling Urinary Catheter	08	53.3	08	53.3	1.000
Nasoenteric Tube	07	46.7	07	46.7	1.000
Oxygen Therapy	09	60.0	09	60.0	1.000
Nasal Oxygen Cannula	04	26.7	05	33.3	0.690
Vaporizer Mask	01	6.7	01	6.7	1.000
Mechanical ventilation	04	26.7	03	20.0	0.666
Mechanical ventilation - A/C^†^ mode	04	26.7	03	20.0	0.666

* Statistical result of Fisher's exact test;

† A/C = Assisted - Controlled

All patients received both types of bath (dry and traditional) following the randomization table, and no damage resulting from the procedures was identified. During the two bathing interventions, the average ambient temperature remained at 22.6ºC (p=0.945). Similarly, there was no significant difference (p=0.925) between the environmental humidity measurements, whose means were 65.4% during the dry bath and 65.3% during the traditional bath.

As regards the time for performance of personal hygiene procedures, the dry bed bath was faster than the traditional bed bath (p<0.001). The dry bath lasted, on average, 20.0 minutes (18.2 - 21.9), and the traditional bath, 30.0 minutes (27.9 - 32.2).

The groups were comparable, since the values of SpO2 (95.7% - dry bath and 95.5% - traditional bath) and RR (19.7 ripm - dry bath and 22.3 ripm - traditional bath) obtained in the first measurement were not statistically different according to analysis by Student’s T test for paired samples (p=0.103 and p=0.859, respectively).

With regard to the effects of the two bed bath types on respiratory parameters, it was found that there was no significant difference between the patients’ SpO2 means in the two procedures (p=0.381), with 94.7% (CI 95 %: 93.8 - 95.5) during the dry bath and 95.2% (95% CI: 94.4 - 96.0) during the traditional bath. Concerning RR, it was observed that, during the traditional bed bath, the RR mean was higher (24.3 ripm; 95% CI: 22.4 - 26.0) and statistically different (p<0.001) from the value found during the dry bath (20.5 ripm; 95% CI: 19.4 - 21.7).

**Figure 2:**
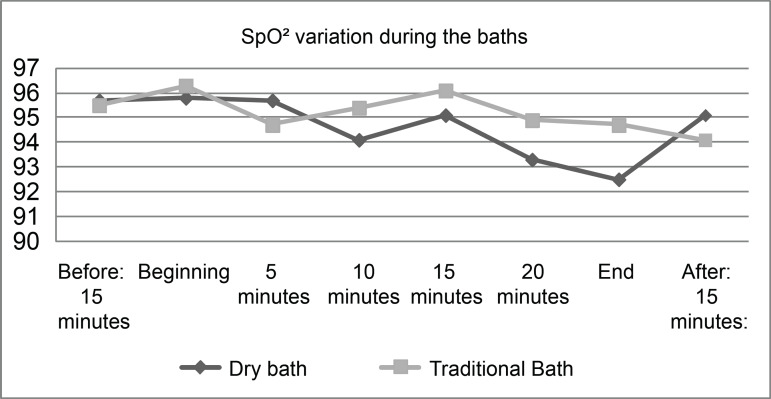
Variation in Transcutaneous Arterial Oxygen Saturation (SpO_2_) in critically ill patients submitted to dry and traditional bed bathing, recorded as a percentage (%), (n=15). Viçosa, MG, Brazil, 2018 ^*^SpO_2_ = Transcutaneous Arterial Oxygen Saturation;

It was found that, during the two bath types, there was a variation in patients’ SpO_2_ values. The highest mean was found at the beginning of each bath, with 95.8% in the dry bath and 96.3% in the traditional bath. Figure 2 shows SpO_2_ variation, in percentage (%), during the two personal hygiene procedures.

**Figure 3:**
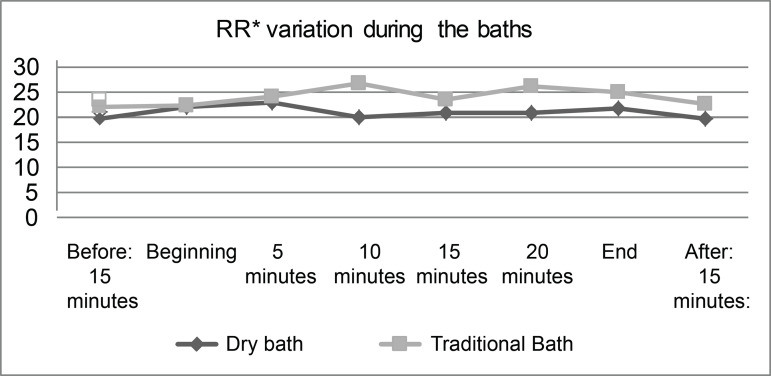
Respiratory Rate (RR) variation in critically ill patients submitted to dry and traditional bed bathing, recorded in ripm, (n=15). Viçosa, MG, Brazil, 2018 ^*^RR = Respiratory Rate

With regard to RR, it was observed that, in the first measurement (15 minutes before each procedure), the lowest RR values were found, which were 19.6 ripm in patients submitted to the dry bath and 22.3 ripm in the traditional bath. In general, during the traditional bath, patients had higher RR means, as shown in Figure 3.

## Discussion

In this study, the sociodemographic profile of critically ill patients was similar to that from other studies conducted in different ICUs, in which there was a predominance of hospitalizations of male, white, elderly patients from sectors within the hospital itself^(^ 21 ^-^ 22 ^)^.

Regarding the cause of hospitalization, there was a predominance of alterations in the respiratory system (46.7%), followed by alterations in the circulatory system (33.3%). The high prevalence of hospitalizations due to respiratory conditions may be related to the effect of seasonality, as data were collected between the months of June and July, the winter period. A study conducted at an ICU in the southern region of Brazil identified circulatory system alterations as the main hospitalization causes (26.3%), while respiratory changes were classified as the fourth cause (11.6%)^(^ 23 ^)^.

In addition to the singularity related to the hospitalization cause, the clinical profile of patients in this study also differed from the findings in the literature. The studied patients were considered to be at lower risk when compared to those admitted to an ICU in São Paulo, in which the majority of patients were sedated, under mechanical ventilation, using a central catheter and receiving some sort of vasoactive medication^(^ 24 ^)^. This difference in the hospitalization profile can be justified by the frequent admission of patients with less complexity due to the absence of a semi-intensive care unit in the city where the studied ICU is located.

All patients were considered dependent on the nursing team for personal hygiene, receiving both types of bed bath (dry and traditional). It was found that the dry bed bath lasted 20 minutes on average, thus being faster than the traditional bed bath (with a mean of 30 minutes). This finding reinforces the results of other international studies, in which the dry bath was also performed in a shorter time^(^ 25 ^-^ 26 ^)^. However, a study developed at an ICU in São Paulo did not report that the dry bath was faster than the traditional bath, since no significant difference was found between the mean duration of each of the two baths. It is believed that one of the reasons why the result was divergent is the fact that dry bathing is a new type of bed bathing performed by professionals who habitually provide the bed bath in the traditional fashion^(^ 18 ^)^.

The time for performance of a bed bath can vary according to the number of professionals involved and the technical skill of each one of them. In this study, all baths were performed by two researchers trained for this purpose. Literature data reinforce that baths that are carried out by only one professional tend to have a longer duration (a mean of 35 minutes) as compared to those performed by two (a mean of 20 minutes)^(^ 8 ^)^.

The longer duration of the traditional bath in relation to that of the dry bath prolongs the exposure of wet patients to the environment and to other risks inherent to the procedure^(^ 5 ^,^ 18 ^,^ 27 ^)^. According to the findings of a systematic review, bed bathing, despite being a routine activity of the nursing team, presents risks to the oxy-hemodynamic stability of critically ill patients when its duration exceeds 20 minutes^(^ 6 ^)^. Thus, the mean duration of the traditional bed bath found in this study (30.0 minutes) places it as a risk factor for these changes.

There is no consensus in the literature regarding the effects of bed bathing on SpO2 in critically ill patients. In a study conducted in Egypt, a significant negative correlation was found between bathing duration in critically ill patients and the level of oxygen saturation^(^ 28 ^)^. However, the authors of a comparative study on dry and traditional bed bathing did not find statistically significant SpO2 changes during the two procedures^(^ 26 ^)^. Similarly, in this investigation, although a variation in SpO2 values was found throughout the procedures, there was no significant difference between the two bed bath types.

Pulse oximetry has been universally used to monitor the respiratory status of critically ill patients in order to provide an early warning of hypoxemia (SpO_2_ <90%), despite the less accurate SpO2 results in the presence of low temperatures^(^ 29 ^-^ 30 ^)^. In this study, the temperature of the ICU environment was a variable controlled by the researchers and, during the evaluation, no patient showed hypoxemia, as the SpO_2_ value was maintained above 90% in all measurements.

In addition to SpO_2_ alterations, RR variation can also be considered as an important indicator of complications in critically ill patients^(^ 15 ^)^. Regarding this parameter, studies have revealed the occurrence of an RR increase in patients submitted to traditional bed bathing^(^ 7 ^-^ 8 ^,^ 26 ^,^ 31 ^)^. The findings in this investigation also showed the negative effects of traditional bed bathing on patients’ RR means, making them higher. It is noteworthy that the RR means in the different types of bed bath were considered statistically different (p<0.001).

The predominance of hospitalizations due to respiratory system alterations (46.7%) and oxygen therapy (60.0%) in this population reinforces the importance of continuous RR monitoring for a safe assessment of patients’ general condition. It is noteworthy that environmental issues, such as low relative humidity, can have a negative influence on patients’ clinical condition and, therefore, they must be constantly observed^(^ 32 ^)^. In this study, humidity in the environment was monitored during all procedures and, although no intervention was made by the researchers, it was kept close to 65% (p=0.925). Nursing professionals must be adequately instructed so that patient safety is assessed before any procedure is performed, with the aim of minimizing the occurrence of adverse events^(^ 33 ^)^.

Bearing in mind that bed bathing may alter patients’ respiratory parameters, during the procedure, nurses must be attentive not only to performing the technique, but also to patients’ behavior. Special attention should be paid to the traditional bed bath, because, although it is a daily practice at ICUs, it was considered a procedure that requires more time to be performed and that presents potential risk for raising RR of critically ill patients. It is essential that nurses perform individualized care, directing their assessment to patients’ manifestations in different ways, whether by verbal contact, by analyzing facial expression and/or by the data obtained with continuous monitoring.

A limitation to this study is the impossibility of guaranteeing the masking of researchers and participants, given the existence of differences between the types of baths to be performed. However, in order to minimize such limitation, the outcomes were collected by an auxiliary researcher who did not participate in the performance of personal hygiene procedures. In addition, as it is a pilot study with a small sample size, the results should be cautiously interpreted, which limits the generalizations of conclusions. Nevertheless, it contributes to clinical practice, as it shows that the dry bath seems to have a shorter duration and, thus, exposes patients to environmental risks resulting from the ICU climate and to those inherent to the procedure itself for a shorter time. The findings in this study serve as useful information to guide the design of future research on the subject.

The next step in this line of research is to conduct other studies, with a representative population sample in order to assess the real effects of dry and traditional bed bathing on variables related to respiratory patterns and hemodynamic behavior of critically ill patients. The data from this study will also help to determine the minimum sample size to detect significant differences between the groups studied. Thus, with new investigations, it will be possible to propose safer nursing practice for fundamental intervention in care provision.

## Conclusion

This study showed that the alternative bed bath method (dry) was considered faster than the traditional bed bath. Regarding the effects generated by the two types of baths on patients’ respiratory patterns, no significant differences were identified between the SpO2 means obtained in each procedure. On the other hand, negative effects of the traditional bed bath were observed for patients’ RR means, making them higher during the procedure.

Such findings reinforce the importance of continuously monitoring patients during their personal hygiene procedures in order to identify significant alterations in clinical conditions, which, despite being transitory, may progress with an increase in patients’ oxygen consumption.
